# Roles of lipocalin-type and hematopoietic prostaglandin D synthases in mouse retinal angiogenesis

**DOI:** 10.1016/j.jlr.2023.100439

**Published:** 2023-09-04

**Authors:** Daiki Horikami, Erika Sekihachi, Keisuke Omori, Yui Kobayashi, Koji Kobayashi, Nanae Nagata, Kaori Kurata, Akiyoshi Uemura, Takahisa Murata

**Affiliations:** 1 Department of Animal Radiology, Graduate School of Agricultural and Life Sciences, The University of Tokyo, Bunkyo-ku, Tokyo, Japan; 2 Department of Retinal Vascular Biology, Nagoya City University Graduate School of Medical Sciences, Mizuho-ku, Nagoya, Japan

**Keywords:** Prostaglandin D_2_, hematopoietic PGD synthase, lipocalin-type PGD synthase, neovascularization, physiologic, pathologic, endothelial cells

## Abstract

Normal angiogenesis is essential for retinal development and maintenance of visual function in the eye, and its abnormality can cause retinopathy and other eye diseases. Prostaglandin D_2_ is an anti-angiogenic lipid mediator produced by lipocalin-type PGD synthase (L-PGDS) or hematopoietic PGD synthase (H-PGDS). However, the exact role of these PGD synthases remains unclear. Therefore, we compared the roles of these synthases in murine retinal angiogenesis under physiological and pathological conditions. On postnatal day (P) 8, the WT murine retina was covered with an elongated vessel. L-PGDS deficiency, but not H-PGDS, reduced the physiological vessel elongation with sprouts increase. L-PGDS expression was observed in endothelial cells and neural cells. In vitro, L-PGDS inhibition increased the hypoxia-induced vascular endothelial growth factor expression in isolated endothelial cells, inhibited by a prostaglandin D_2_ metabolite, 15-deoxy-Δ^12,14^ -PGJ_2_ (15d-PGJ_2_) treatment. Pericyte depletion, using antiplatelet-derived growth factor receptor-β antibody, caused retinal hemorrhage with vessel elongation impairment and macrophage infiltration in the WT P8 retina. H-PGDS deficiency promoted hemorrhage but inhibited the impairment of vessel elongation, while L-PGDS did not. In the pericyte-depleted WT retina, H-PGDS was expressed in the infiltrated macrophages. Deficiency of the D prostanoid receptor also inhibited the vessel elongation impairment. These results suggest the endogenous role of L-PGDS signaling in physiological angiogenesis and that of H-PGDS/D prostanoid 1 signaling in pathological angiogenesis.

Angiogenesis is a process in which new blood vessels branch from pre-existing vessels and is crucial for maintaining homeostasis under physiological and pathological conditions (1). Physiological angiogenesis is observed in tissue development and repair, while pathological angiogenesis is observed in chronic inflammation. In the eye, normal angiogenesis is also indispensable for retinal development and maintenance of visual function, of which abnormality can lead to some eye diseases, including retinopathy and macular degeneration.

The mechanisms underlying physiological and pathological angiogenesis have been well investigated. During angiogenesis, hypoxic stress initially stimulates the expression of several pro-angiogenic factors, including vascular endothelial growth factor-A (VEGF-A), fibroblast growth factor-2 (FGF-2), and epidermal growth factor (EGF) (2, 3, 4). VEGF-A causes endothelial cell (EC) migration via VEGF receptors (VEGFRs). The migrated ECs, called “tip cells,” promote new sprout formation (2). The ECs neighboring tip cells, called “stalk cells,” proliferate and form the vascular lumen. FGF-2 also promoted the migration and proliferation of ECs (3). After vascular lumen formation, ECs secrete platelet-derived growth factor (PDGF), which recruits pericytes (PCs) via PDGF receptor β (PDGFRβ). For physiological angiogenesis, recruited PCs are important to cover ECs and stabilize the vascular structures (5, 6). On the other hand, in pathological angiogenesis, excessive inflammation stimulates the production of growth factors and cytokines, including interleukin-1β (IL-1β), tumor necrosis factor α (TNFα), and stromal cell-derived factor 1α (SDF1α, also known as CXCL12) (7, 8, 9). These pro-inflammatory mediators induce chaotic and nondirectional proliferation of ECs without stabilization by PCs. Unstabilized ECs lead to vascular hyperpermeability and macrophage infiltration, which results in further inflammation. These abnormally formed blood vessels further accelerate disease progression.

PGD_2_ is one of the major PGs synthesized by lipocalin-type PGD synthase (L-PGDS) and hematopoietic PGD synthase (H-PGDS). L-PGDS is expressed in the central nervous system, by astrocytes and oligodendrocytes, under the regulation of NF-E2-related factor 2 and activator protein (AP)-1 (10, 11). H-PGDS is expressed in inflammatory cells, such as mast cells, megakaryocytes, and macrophages under the regulation of Ras guanyl nucleotide-releasing protein 4, organic cation transporter-1, and AP-2 (12, 13). The synthesized PGD_2_ works as a ligand of two distinct G protein-coupled receptors: D prostanoid (DP) 1 and DP2. Our group previously showed that gene deficiency of both L-PGDS and H-PGDS increased vascular permeability and angiogenesis in murine lung carcinoma models (14, 15). Although these results highlight the anti-angiogenic effect of PGD_2_ signaling in tumor growth, the difference and/or significance of these synthases in angiogenesis remain unclear.

In the present study, we aimed to compare the role of two PGD synthases, L-PGDS and H-PGDS, in angiogenesis by focusing on two murine retinal angiogenesis: neonatal physiological angiogenesis and PC depletion-induced pathological angiogenesis.

## Materials and Methods

### Mice

All animal experiments were approved by the Institutional Animal Care and Use Committee of The University of Tokyo (Approved No. P23-066). Mice were housed in 12-12 h light-dark cycles. C57BL/6J WT mice were purchased (CLEA Japan Inc., Tokyo, Japan). L-PGDS-deficient (*L-pgds*^−/−^) mice, H-PGDS-deficient (*H-pgds*^−/−^) mice, DP1-deficient (*Dp1*^−/−^) mice, and DP2-deficient (*Dp2*^−/−^) mice were generated as previously described (16, 17, 18, 19). All mice were C57BL/6J background.

P1 neonatal mice were administrated with rat anti-PDGFRβ antibody (APB5, intraperitoneally, 50 μg/50 μl in PBS; purified as previously described (6)). The control mice were administrated with only PBS. After the administration, mice were euthanized by cervical dislocation, and the eyes were excised and analyzed for further experiments.

### Morphological analysis of mice retina

Excised mice eyes of postnatal day (P) 4, P8, and P14 were fixed with 4% paraformaldehyde (PFA) in PBS for 30 min. After the fixation, retinal cups were dissected and fixed again with 4% PFA overnight.

The fixed cups were blocked with 5% bovine serum albumin (ProSpec-Tany TechnoGene Ltd., Rehovot, Israel), permeabilized with 0.1% Triton X-100 in PBS for 1 h, and then incubated overnight at 4°C with primary antibodies; rabbit anti-desmin antibody (1:200; ab15200; Abcam, Cambridge, UK), rabbit anti-fibrinogen antibody (1:500; A0080; Dako, Glostrup, Denmark), rat anti-F4/80 antibody (1:200; Cl:A3-1, MCA497APCT; Bio-Rad, CA), rat anti-CD68 antibody (1:200; FA-11, MCA1957; AbD Serotec, Kidlington, UK), and rabbit anti-H-PGDS antibody (1:200; 10004348, Invitrogen, CA). The cups were then incubated for 2 h at room temperature with Alexa Fluor 594–conjugated isolectin B4 (1:200; I21413; Invitrogen) and secondary antibodies; Alexa Fluor 488 anti-rabbit antibody (1:500; A11008; Invitrogen), Alexa Fluor 488 anti-rat antibody (1:500; A11006; Invitrogen) in 0.1% Triton X-100 in PBS. After the incubation, the cups were cut and opened within four slices and observed using a Nikon Eclipse Ti microscope and C1 confocal system (Nikon, Tokyo, Japan). The images were analyzed with NIS-Elements D software (Nikon).

The vessel elongation was calculated by averaging the distances from the optic nerve to the end of the angiogenic front in each slice. The number of sprouts was also calculated by averaging the counted sprouts number in the angiogenic front of each slice. Finally, the number of macrophages was calculated from the averaged counts in four 300 × 300 μm fields in the angiogenic area.

### Immunostaining of mice retinal cross-section

P8 retinal cups were excised and fixed with 4% PFA for 24 h. The fixed cups were embedded in paraffin and sliced into cross-sections (4 μm). The sections were blocked, permeabilized, and incubated at 4°C overnight with rat monoclonal anti-L-PGDS antibody (1:500; raised and purified by Eguchi *et al.* (16)), rabbit anti-PGP9.5 antibody (1:200; ADI-905-520-1; Enzo Life Sciences, NY), followed by the incubation with Alexa Fluor 594–conjugated isolectin B4 and secondary antibody. Fluorescent and confocal microscopic images were captured as described above.

### Measurement of prostanoids

P4 and P8 retinal cups were excised, snap-frozen, and homogenized in 100 μl PBS. Then, 50 μl of homogenized samples were mixed with 50 μl MeOH, 10 μl formic acid, and 900 μl DW. After the centrifugation, 800 μl of supernatants were added with internal standards, PGE_2_-d4 (314010; Cayman chemical, MA) and PGD_2_-d4 (312010; Cayman chemical) and then purified by solid phase extraction cartridge (OASIS HLB μElution plate, Waters, Massachusetts). The samples were eluted by 50 μl MeOH and loaded to high-performance liquid chromatography (Nexera 2; Shimadzu, Kyoto, Japan) equipped with a mass spectrometer (LCMS-8060; Shimadzu, Kyoto, Japan). We used Inertsil ODS-HL 2.1×100 mm 1.9 um (5020-87344; GL Science, Tokyo, Japan) for the liquid chromatographic separation and 0.1% formic acid and acetonitrile for a mobile phase. The concentration of PGE_2_ and PGD_2_ were normalized by the internal standards and calculated by the control, PGE_2_ (14,010; Cayman chemical) and PGD_2_ (12,010; Cayman chemical).

### Real-time PCR of mice retina

Retinal cups were excised from the eyes of P4 and P8 mice. Total RNA was extracted using TRI-Reagent (Molecular Research Center, OH). The complementary DNA (cDNA) was obtained by reverse transcription using a Random 9-mer primer (TOYOBO, Tokyo, Japan) and ReverTra Ace (TOYOBO) at 30°C for 10 min, 42°C for 1 h, and 99°C for 5 min according to the manufacturer’s protocol. Then, the cDNA was amplified for 45 cycles of 95°C for 15 s and 59°C for 1 min, using Platinum SYBR Green qPCR SuperMix-UDG (Thermo Fisher Scientific, MA) and AriaMx Real-Time PCR System (Agilent Technologies, CA). The mRNA expression levels were quantitated with the ΔΔCt method, using 18S-rRNA levels as internal controls. The list of primer sequences is shown in supplemental Table S1.

### Scoring retina

We scored excised APB5-treated retinal cups as described previously (8), which were defined as follows: grade 0, retina with no hemorrhage or edema; grade 1, local hemorrhage; grade 2, hemorrhage and/or mild edema; grade 3, edema in up to one-half of the retina; and grade 4, the collapse of the retina.

### Cell culture and real-time PCR of HUVECs

Human umbilical vein endothelial cells (HUVECs; passages, 5–8; Lonza, Basel, Swiss) were cultured in Endothelial Growth Medium-2 (Lonza) containing 10% fetal bovine serum under normoxia (5% CO_2_ and 20% O_2_). HUVECs were starved for 4 h in Endothelial Basal Medium-2 (Lonza) containing 2% fetal bovine serum under normoxia and then incubated for 6 h under normoxia (5% CO_2_ and 20%–21% O_2_) or hypoxia (5% CO_2_ and 5% O_2_). In addition, an L-PGDS inhibitor (AT-56, 100 μM; Cayman chemical) and 15-deoxy-Δ^12,14^ PGJ_2_ (15d-PGJ_2_, 3 μM; Cayman chemical) were pretreated 5 min before the incubation.

The total RNA of HUVECs was extracted, and cDNA was obtained by reverse transcription. The cDNA was amplified by using specific primers (supplemental Table S2). The mRNA expression levels were quantitated with the ΔΔCt method, using 18S-rRNA levels as internal controls.

### Statistical analysis

**Fig. 1:**
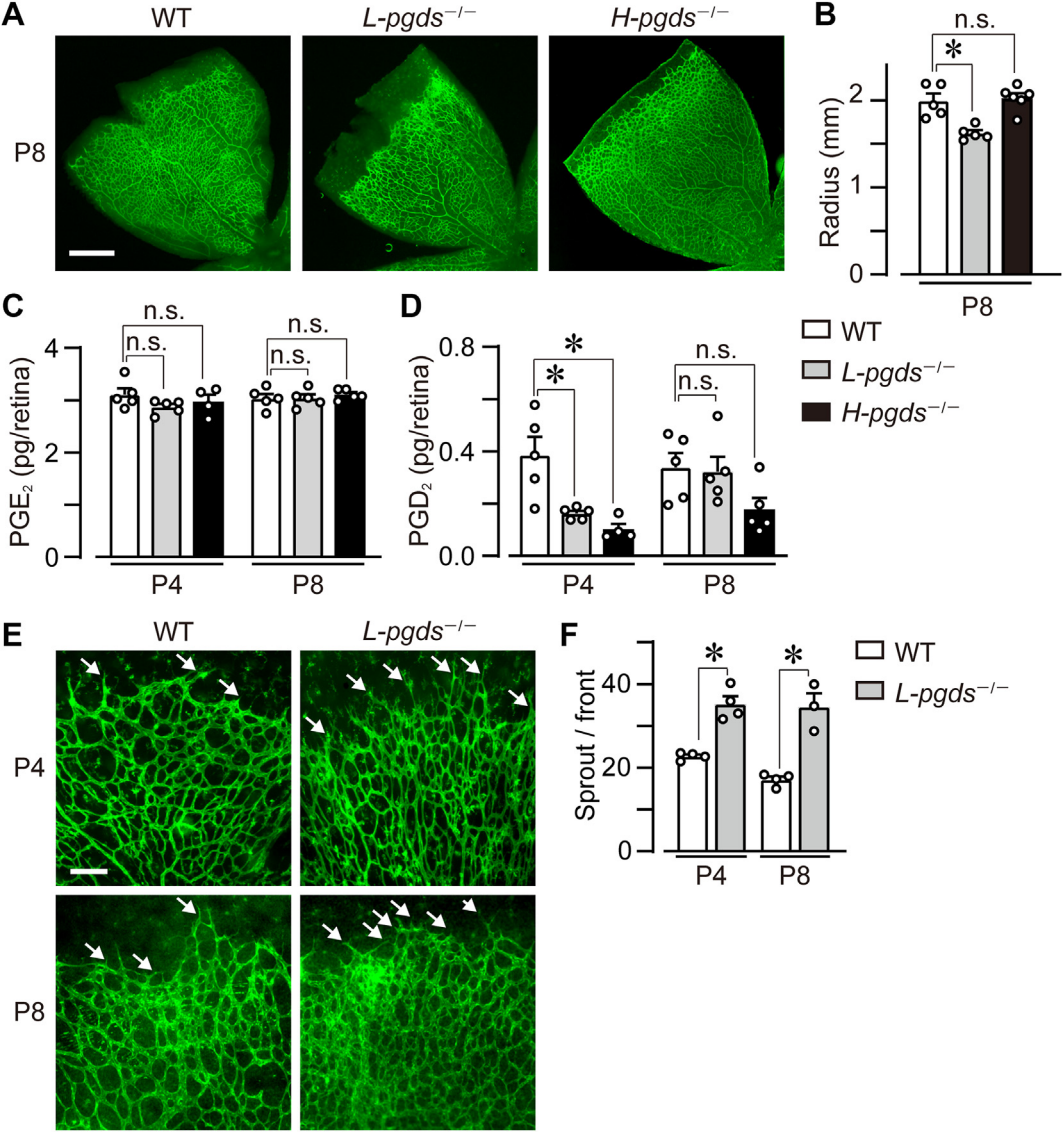
L-PGDS deficiency inhibited vessel elongation in the neonatal retinal angiogenesis. P4 and P8 retina of WT, *L-pgds*^−/−^, and *H-pgds*^−/−^ mice were excised, and ECs were stained with isolectin B4 (green). A: Representative picture of the retina (n = 6–8). Scale bar, 500 μm. B: The summary of vessel elongation (n = 5–6). C: The summary of PGE_2_ level in P4 and P8 retina (n = 4–5). D: The summary of PGD_2_ level in P4 and P8 retina (n = 4–5). E: Representative pictures of the front of the retinal vessel (n = 3–4). Scale bar, 100 μm. Arrows indicate angiogenic sprouts. F: The summary of the number of sprout/front (n = 3–4). Data were represented as mean ± SEM. ∗*P* < 0.05. n.s., not significant. EC, endothelial cell; L-PGDS, lipocalin-type PGD synthase; H-PGDS, hematopoietic PGD synthase; PGD2, prostaglandin D2.

All data are shown as mean ± SEM. The normality of data was analyzed using the Shapiro-Wilk test (α = 0.05), and equal variance was analyzed using Bartlett’s test (α = 0.05) using BellCurve for Excel (Social Survey Research Information Co., Tokyo, Japan). The data which failed the normality or equal variance test was treated as nonparametric data (Fig. 1C-P4, 1E-P4, 2C, 2D, 2E, 2F, 2G-P8, 2H, 2I-P4, 4B, 5B, 6B, S2C-Artery, S3B, S3B, S3C, and S3D). The statistical difference was determined by Student’s *t* test or one-way ANOVA with Tukey’s test for parametric analysis and Mann-Whitney U test or Kruskal-Wallis test with Steel-Dwass test for nonparametric data. Statistical significance was determined when *P*-value was less than 0.05.

## Results

### L-PGDS deficiency inhibited vessel elongation in the neonatal retinal angiogenesis

In neonatal mice, the retinal vessel elongates from the optic papilla towards the edge of the retina from P0 (20). This phenomenon is utilized as a physiological angiogenesis model by staining ECs with isolectin B4. Firstly, we observed neonatal retinal vessels in WT, L-PGDS-deficient (*L-pgds*^−/−^), and H-PGDS-deficient (*H-pgds*^−/−^) mice. On P8, the retinal vessel elongated to the end of the tissue in the WT mice (Fig. 1A, left panel). L-PGDS deficiency (middle panel), but not H-PGDS deficiency (right panel), significantly decreased the vessel elongation on P8 (Fig. 1B; WT, 2.0 ± 0.1 mm; *L-pgds*^−/−^, 1.6 ± 0.1 mm; *H-pgds*^−/−^, 2.0 ± 0.1 mm). In the WT retina, the retinal vessel elongated to the middle of the tissue at P4 (supplemental Fig. S1A, upper panels) and sprouted into the deep layer on P14 (lower panels). L-PGDS deficiency slightly, but not significantly, decreased vessel elongation at P4 (supplemental Fig. S1B) and did not affect it at P14.

We measured the level of PGE_2_ (one of major PGs as a control) and PGD_2_ in P4 and P8 retina using LC/MS system. L-PGDS or H-PGDS deficiency did not affect the level of PGE_2_ in P4 and P8 retina (Fig. 1C). On the other hand, both L-PGDS and H-PGDS deficiency significantly decreased the level of retinal PGD_2_ in P4 mice, not in P8 (Fig. 1D). Although we also tried to measure the retinal level of Δ12-PGJ_2_ and 15d-PGJ_2_, we could not detect them in P4 and P8 retina (data not shown).

Next, we investigated the angiogenesis front of the retinal vessels. As shown in Fig. 1E, F, L-PGDS deficiency significantly increased the number of sprouts on both P4 and P8. We also assessed the diameter of the retinal vein and artery near the optic papilla as indices of vessel abnormalities (supplemental Fig. S1C). However, these parameters did not differ between WT, *L-pgds*^−/−^, or *H-pgds*^−/−^ mice (supplemental Fig. S1D).

These results suggest that L-PGDS, but not H-PGDS, was required for vessel elongation and sprouts redocumentation in the neonatal retinal angiogenesis.

### Endothelial and neural L-PGDS reduced the expressions of pro-angiogenic factors

**Fig. 2:**
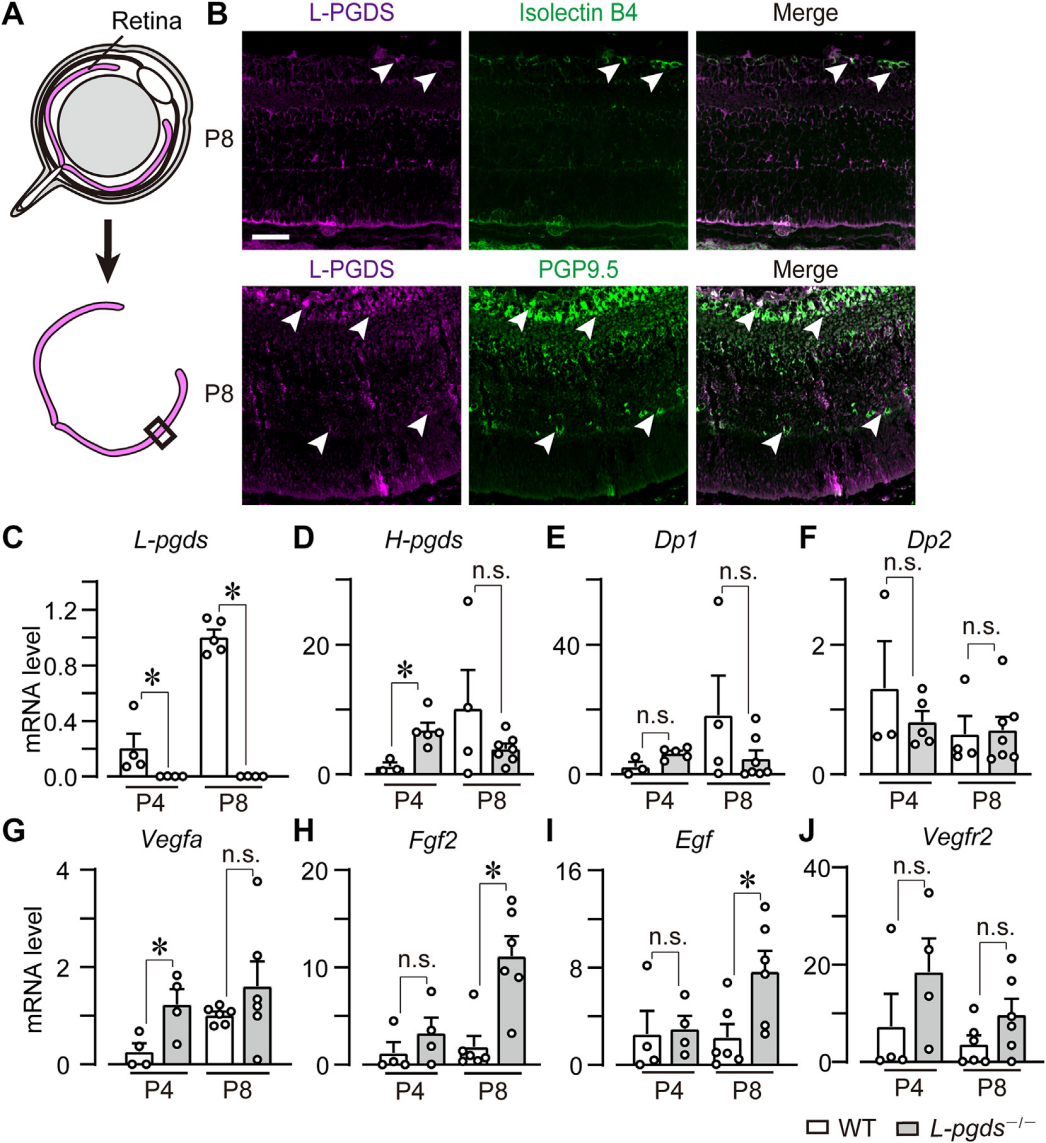
Endothelial and neural L-PGDS reduced the expressions of pro-angiogenic factors. P4 and P8 retina of WT and *L-pgds*^−/−^ mice were excised and used for immunostaining and real-time PCR analysis. A: The illustration of the method of the cross-section of the retina. B: Representative pictures of the cross-sections of WT mice retina. Immunostaining of L-PGDS (magenta, n = 4–5) with isolectin B4 (green, upper panels) or PGP9.5 (green, lower panels). Scale bar, 50 μm. C–G: The summary of mRNA level of (C) L-PGDS (*L-pgds*, n = 4–5), (D) H-PGDS (*H-pgds*, n = 4–7), (E) DP1 (*Dp1*, n = 4–7), (F) DP2 (*Dp2*, n = 4–7), (G) VEGF-A (*Vegfa*, n = 4–6), (H) FGF-2 (*Fgf2*, n = 4–6), (I) EGF (*Egf*, n = 4–6), and (J) VEGFR2 (*Vegfr2*, n = 4–6). Data were represented as mean ± SEM. ∗*P* < 0.05. n.s., not significant. DP, D prostanoid; EGF, epidermal growth factor; FGF, fibroblast growth factor; H-PGDS, hematopoietic PGD synthase; VEGF, vascular endothelial growth factor; L-PGDS, lipocalin-type PGD synthase; VEGFR, VEGF receptor.

To clarify the L-PGDS-producing cell, immunofluorescent staining was performed in the cross sections of the P8 WT retina (Fig. 2A). L-PGDS was localized in isolectin B4-positive ECs (Fig. 2B, upper panels) and PGP9.5-positive neural cells (lower panels). The retina is divided into several neuron cell layers. L-PGDS expression was observed in the ganglion cells and inner nuclear cell layers. In addition, we confirmed the existence of L-PGDS (L-pgds) mRNA in the P4 and P8 retinas, which were suppressed by L-PGDS deficiency (Fig. 2C). In addition, we also measured the mRNA expression of PGD_2_-related genes: H-PGDS, DP1, and DP2. L-PGDS deficiency significantly increased the mRNA level of H-PGDS in P4 retina, not in P8 retina (*H-pgds*, Fig. 2D). On the other hand, L-PGDS deficiency did not affect the mRNA expression of DP1 (*Dp1*, Fig. 2E) and DP2 (*Dp2*, Fig. 2F).

We further investigated the role of L-PGDS in the mRNA levels of vascular growth factors. L-PGDS deficiency significantly increased the mRNA level of VEGF-A (*Vegfa*, Fig. 2G) but not FGF-2 (*Fgf2*, Fig. 2H), EGF (*Fgf*, Fig. 2I), or VEGFR2 (*Kdr*, Fig. 2J) in the P4 retina. In the P8 retina, L-PGDS deficiency significantly increased the mRNA levels of FGF-2 and EGF but not VEGF-A or VEGFR2.

These results suggest that L-PGDS signaling reduced excessive expression of pro-angiogenic factors, which probably leads to chaotic and dysregulated vessel elongation at P8.

### DP1 and DP2 were not involved in L-PGDS signaling in neonatal retinal angiogenesis

**Fig. 3:**
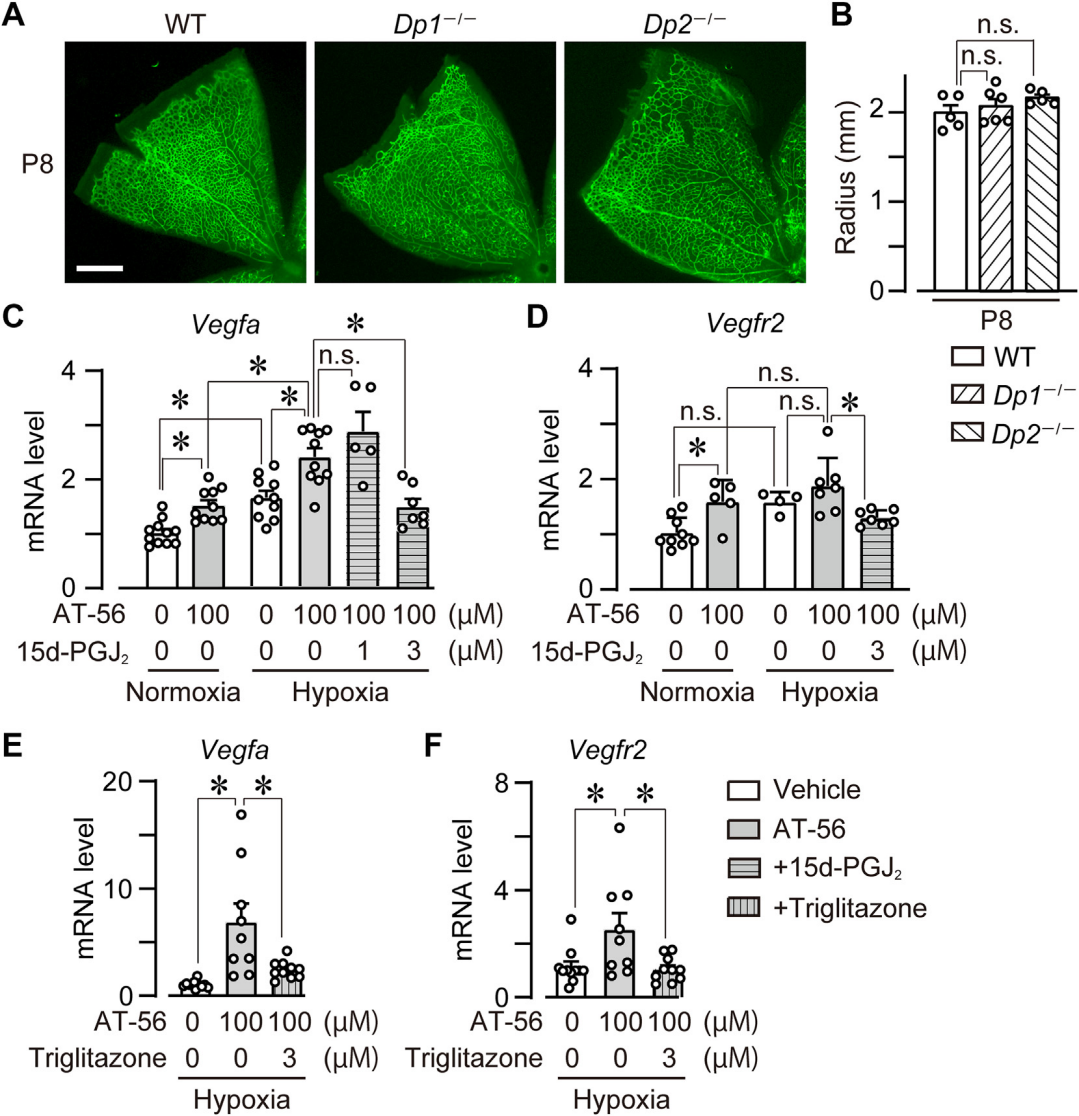
DP1 and DP2 were not involved in L-PGDS signaling in neonatal retinal angiogenesis. A and B: P4 and P8 retina of WT, *Dp1*^*−/−*^, and *Dp2*^*−/−*^ mice were excised, and ECs were stained with isolectin B4 (green). A: Representative picture of the retina (n = 5–6). Scale bar, 500 μm. B: The summary of vessel elongation (n = 5–6). The data of the P8 WT group is identical to Fig. 1B. C and D: HUVECs were treated with AT-56 (100 μM) and 15d-PGJ_2_ (1 or 3 μM) for 6 h under normoxia or hypoxia. The summary of mRNA level of (C) VEGF-A (*Vegfa*, n = 5–11) and (D) VEGFR2 (Vegfr2, n = 4–9). E and F: HUVECs were treated with AT-56 (100 μM) and troglitazone (3 μM) for 6 h under hypoxia. The summary of mRNA level of (E) VEGF-A (*Vegfa*, n = 9–11) and (F) VEGFR2 (*Vegfr2*, n = 9–11). Data were represented as mean ± SEM. ∗*P* < 0.05. n.s., not significant. DP, D prostanoid; EC, endothelial cell; HUVEC, human umbilical vein endothelial cell; L-PGDS, lipocalin-type PGD synthase; VEGFR, VEGF receptor; VEGF, vascular endothelial growth factor.

Since L-PGDS deficiency decreased retinal PGD_2_, we investigated the role of PGD_2_ receptors, DP1 and DP2, in vessel elongation of P8 retina. However, compared with WT mice, DP1 receptor-deficient (*Dp1*^−/−^) mice and DP2 receptor-deficient (*Dp2*^−/−^) mice exhibited no change of vessel elongation (Fig. 3A, B; WT, 2.0 ± 0.1 mm; *Dp1*^−/−^, 2.1 ± 0.1 mm; *Dp2*^−/−^, 2.1 ± 0.1 mm). These gene deficiencies also did not influence the vessel diameters and the number of branch points (data not shown).

PGD_2_ is known to be metabolized to 15d-PGJ_2_, an endogenous ligand of peroxisome proliferator-activated receptor (PPAR)γ. We here investigated the effect of 15d-PGJ_2_ on L-PGDS signaling using isolated HUVECs. HUVECs express L-PGDS in response to various stimuli. Under both normoxia (20%–21% O_2_) and hypoxia (5% O_2_), treatment with an L-PGDS inhibitor (AT-56, 100 μM, 6 h) to HUVECs significantly increased the mRNA level of VEGF-A (*Vegfa*) (11, 14). This increase was significantly higher in hypoxia group (Fig. 3C). The pretreatment with 15d-PGJ_2_ (3 μM, not 1 μM) suppressed this AT-56-induced increase of VEGF-A expression. We also found that, under only normoxia, L-PGDS inhibition increased the mRNA level of VEGFR2 (Vegfr2) significantly (Fig. 3D). Under the hypoxic condition, the pretreatment with 15d-PGJ_2_ (3 μM) significantly reduced the VEGFR2 expression. In addition, the treatment with troglitazone, an independent PPARγ agonist, significantly reduced the expression of VEGFA (Fig. 3E) and VEGFR2 (Fig. 3F) in hypoxic HUVEC like the 15d-PGJ_2_ treatment.

This result indicates that DP1 and DP2 are not involved in L-PGDS-induced physiological angiogenesis, in which 15d-PGJ_2_/PPARγ axis probably involved.

### H-PGDS deficiency aggravated symptoms in PC depletion-induced retinopathy model

**Fig. 4:**
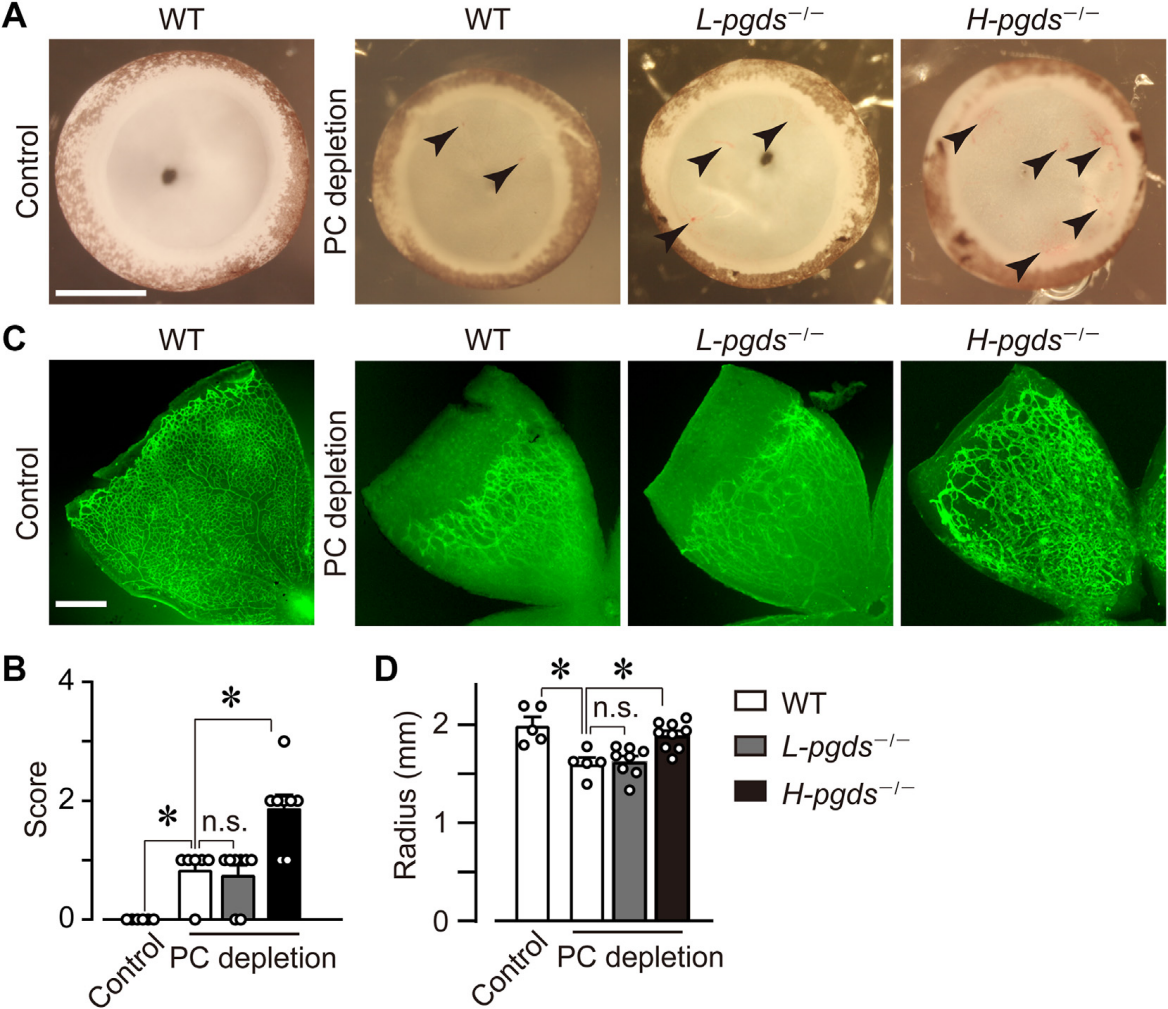
H-PGDS deficiency aggravated symptoms in PC depletion-induced retinopathy model. After the PC depletion on P1 WT, *L-pgds*^−/−^, and *H-pgds*^−/−^ mice, the P8 retina was excised, observed, and stained with isolectin B4 (green). A: Representative picture of the excised retina (n = 6–8). Scale bar, 500 μm. Arrowheads indicate the hemorrhage site. B: The summary of retina grading scores (n = 6–8). C: Representative picture of the retina (n = 5–9). Scale bar, 500 μm. D: The summary of vessel elongation (n = 5–9). The data of the WT control group is identical to Fig. 1B WT P8 group. Data were represented as mean ± SEM. ∗*P* < 0.05. n.s., not significant. H-PGDS, hematopoietic PGD synthase; L-PGDS, lipocalin-type PGD synthase;PC, pericyte.

We then investigated the roles of L-PGDS and H-PGDS in pathological retinal angiogenesis using PC depletion-induced retinopathy model. The administration of an anti-PDGFRβ antibody, APB5, depletes PCs in developing mouse retinas. This PC depletion leads to clinical symptoms of human diabetic retinopathy, including blood-retina barrier breakdown, retinal edema, and hemorrhage (6). In the vehicle-treated P8 WT retina, retinal isolectin B4-positive ECs were covered by desmin-positive PCs (supplemental Fig. S2A, left panel). The administration of APB5 (50 μg in 50 μl PBS/head at P1, intraperitoneally) depleted PCs around ECs in P8 WT, *L-pgds*^*−/−*^, and *H-pgds*^−/−^ mice (supplemental Fig. S2A; right panels). The administration of APB5 also caused bleeding (black arrowheads) inside the retinal cups in P8 WT mice (Fig. 4A). L-PGDS deficiency did not differ from the bleeding in WT, while H-PGDS deficiency seemed to be aggravated the bleeding in APB5-treated retinas. We also quantified “retinal grading score” that represents the level of retinal edema and hemorrhage. PC depletion significantly increased the score in P8 WT (Fig. 4B), which H-PGDS deficiency but not L-PGDS significantly increased.

The APB5-induced PC depletion is also known to causes distortion, shortness, and dilation of retinal vessels (6). These are characteristic remark of pathological angiogenesis. PC depletion in WT mice induced the decrease of vascular elongation accompanied by impaired vascular structure (Fig. 4C, D; control WT, 2.0 ± 0.1 mm; PC-depleted WT, 1.6 ± 0.1 mm). Contrary to the results of the retinal grading score, H-PGDS deficiency inhibited the APB5-induced shortage of vascular elongation (PC-depleted *H-pgds*^*−/−*^ mice, 1.9 ± 0.1 mm). L-PGDS deficiency did not affect the vessel elongation (PC-depleted *L-pgds*^*−/−*^ mice, 1.6 ± 0.1 mm). In addition, in WT mice, APB5 administration tended to increase the diameter of the vein, but not the artery (supplemental Fig. S2B, C). Neither L-PGDS nor H-PGDS deficiency affected the diameter of the veins or arteries.

These results suggest that H-PGDS, but not L-PGDS, is involved in PC depletion-induced pathological angiogenesis.

### H-PGDS is located in the macrophage of PC-depleted P8 retina

**Fig. 5:**
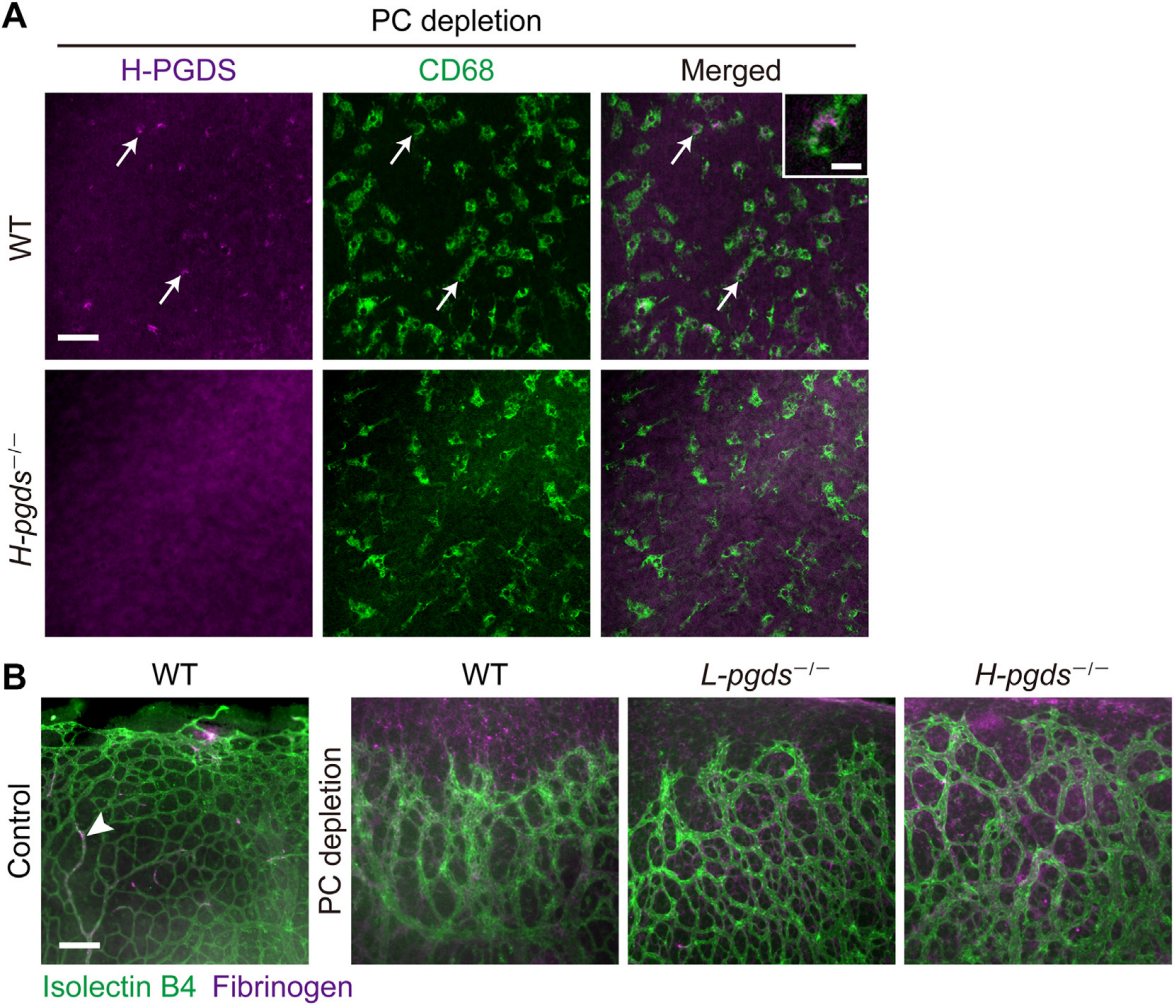
H-PGDS was located in macrophage of PC-depleted P8 retina. After the PC depletion on P1 WT and *H-pgds*^−/−^ mice, the P8 retina was excised and used for immunostaining and real-time PCR analysis. A: Representative pictures of immunostaining of H-PGDS (magenta, left panels) with CD68 (green, middle panels) (n = 3–4). Scale bar, 25 μm. Upper right panel shows high power field of H-PGDS-expressing CD68-positive macrophage. Scale bar, 5 μm. B: Representative pictures of the front of the retinal vessel stained with isolectin B4 (green) and fibrinogen (magenta, n = 3–4). Scale bar, 100 μm. The arrowhead indicates fibrinogen inside the vessel. H-PGDS, hematopoietic PGD synthase; PC, pericyte.

We next investigated the H-PGDS-expressing cell using en-face immunofluorescence staining. In PC-depleted WT retinas, H-PGDS expression was observed in CD68-positive macrophages (Fig. 5A, upper panels, white arrows). We confirmed that H-PGDS expression was not observed in H-PGDS-deficient retina (lower panels).

Several studies have shown that PC depletion causes inflammation indicated as vascular hyperpermeability and macrophage infiltration (6, 8). We tried to clarify the effect of H-PGDS on the PC depletion-induced inflammatory responses. In control P8 WT retina, en-face immunostaining showed that fibrinogen (plasma protein, green) was observed inside the retinal vessel (Fig. 5B, left panel, white arrowhead). PC depletion caused fibrinogen extravasation, representing vascular hyperpermeability (right panels). Compared to WT, fibrinogen extravasation seemed to be stronger in *H-pgds*^−/−^ retinas but not in *L-pgds*^−/−^. In WT retinas, PC depletion also caused F4/80-positive macrophage infiltration (supplemental Fig. S3A, B, left panel). However, H-PGDS or L-PGDS deficiency did not affect the number of macrophage (right panels).

We next investigated the effect of H-PGDS deficiency on the mRNA expression of inflammatory cytokines. These cytokines play important role in the pathological angiogenesis of PC depletion-induced retinopathy model (6, 8). In WT, APB5 administration increased the mRNA expression levels of TNFα (*Tnfa*, supplemental Fig. S3C; *P* = 0.02) significantly, but not IL-1β (*Il1b*, supplemental Fig. S3D; *P* = 0.17) and SDF1α (*Cxcl12*, supplemental Fig. S3E; *P* = 0.10) slightly. H-PGDS deficiency slightly, but not significantly, increased the expression level of IL-1β (*P* = 0.20). H-PGDS deficiency did not affect the expression of TNFα (*P* = 0.96) and SDF1α (*P* = 0.96).

These results indicate that H-PGDS is expressed in macrophage, which inhibited vessel elongation without affecting macrophage infiltration.

### DP1 receptor deficiency inhibited PC depletion-induced inhibition of vessel elongation

**Fig. 6:**
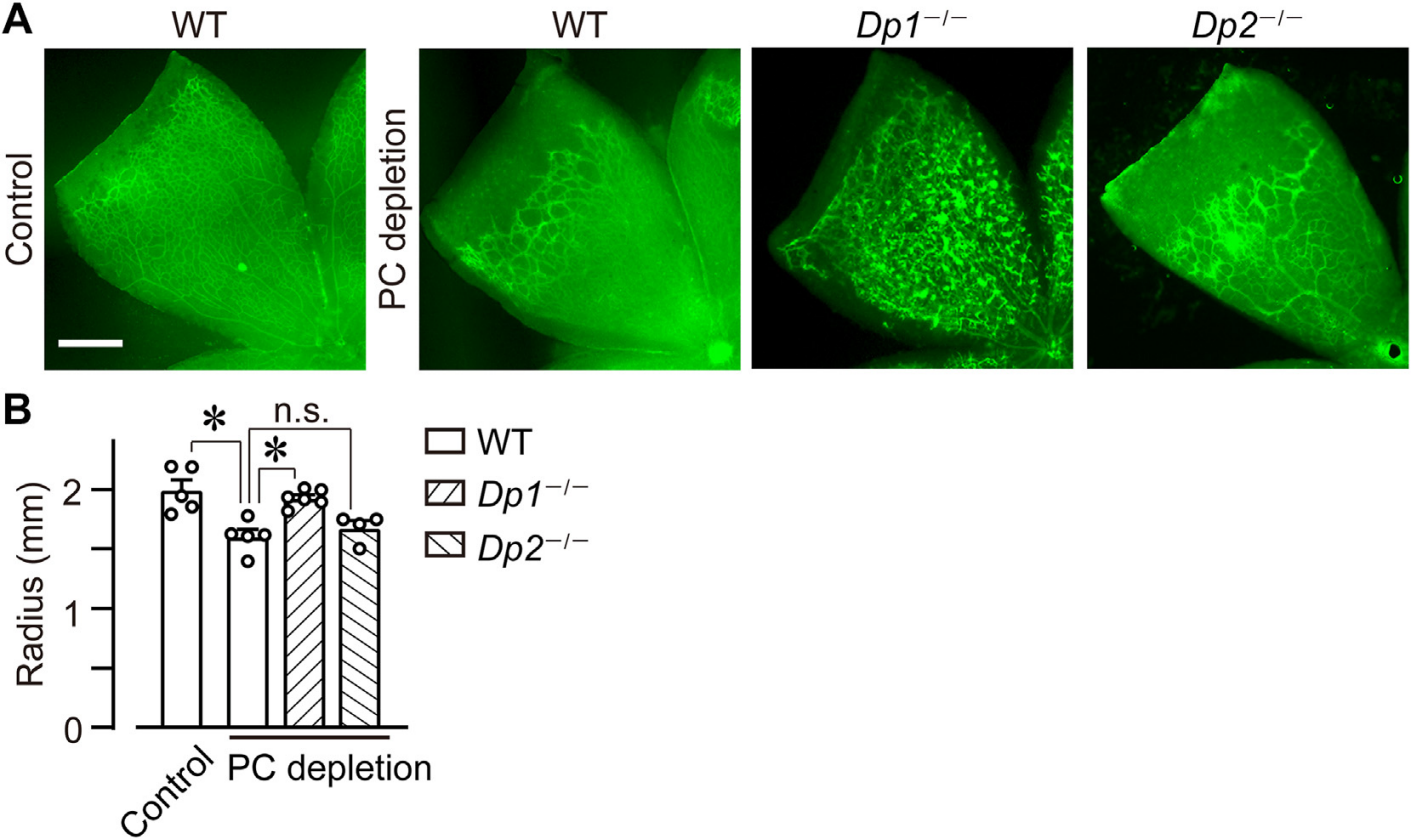
DP1 receptor deficiency inhibited PC depletion-induced inhibition of vessel elongation. After the PC depletion on P1 WT, *Dp1*^*−/−*^, and *Dp2*^*−/−*^ mice, the P8 retina was excised and stained with isolectin B4 (green). A: Representative picture of the retina (n = 4–8). Scale bar, 500 μm. B: The summary of vessel elongation (n = 4–6). The data of the WT control group is identical to Fig. 1B WT P8 group. The data of the PC-depleted WT group are identical to Fig. 4B WT group. Data were represented as mean ± SEM. ∗*P* < 0.05. n.s., not significant. DP, D prostanoid; PC, pericyte.

Finally, we examined the downstream signal of H-PGDS in the PC depletion-induced decrease of vascular elongation by utilizing gene deficiency of PGD_2_ receptors, DP1 and DP2. Compared with the PC-depleted WT mice, gene deficiency of DP1 but not that of DP2 inhibited the decrease in vascular elongation (Fig. 6A, B; WT mice, 1.6 ± 0.1 mm; *Dp1*^*−/−*^ mice, 1.9 ± 0.1 mm; and *Dp2*^*−/−*^ mice, 1.6 ± 0.1 mm). The radius of retinal vessel in PC-depleted *Dp1*^*−/−*^ mice was like the WT control group (2.0 ± 0.1 mm) and *H-pgds1*^*−/−*^ mice (*H-pgds*^*−/−*^ mice, 1.9 ± 0.1 mm).

This result suggests that DP1 was probably the downstream signaling of H-PGDS in the PC depletion-induced pathological angiogenesis.

## Discussion

In the present study, we investigated the role of L-PGDS and H-PGDS in physiological and pathological angiogenesis using mouse retina models. We detected L-PGDS expression in ECs and neural cells of the neonatal retina, while H-PGDS was expressed in macrophages in PC-depleted retina. In neonatal retinal angiogenesis model, L-PGDS deficiency, but not H-PGDS, DP1, or DP2 deficiency, reduced vessel elongation with the excessive transcription of pro-angiogenic factors. While in PC depletion-induced retinopathy model, H-PGDS and DP1 deficiency, but not L-PGDS or DP2, inhibited the APB5-induced vessel elongation decrease but aggravated retinal grading score.

It is known that pro-angiogenic factors such as VEGF, FGF-2, and EGF play a crucial role in retinal angiogenesis. In murine neonatal retinal angiogenesis, tip cells at the angiogenic front ECs migrate according to the VEGF-A gradient from astrocyte (21). The costimulation with VEGF-A and FGF-2 on Matrigel plug-in mice abdomen synergistically promotes angiogenesis (22). Conversely, the excessive secretion of pro-angiogenic factors and/or excessive sprout formation impairs normal angiogenesis. Overexpression of VEGF induced chaotic sprout formation, resulting in delayed vessel elongation (23). Kim *et al.* also showed that deficiencies of large tumor suppressor half increased tip cell number, leading to decreased vessel elongation and dysregulated angiogenesis (24). Thus, regulating adequate pro-angiogenic factors is essential for neonatal retinal angiogenesis.

In this study, we found that L-PGDS deficiency, but not H-PGDS deficiency, impaired normal retinal angiogenesis. L-PGDS deficiency also increased the number of sprouts with partial VEGF, FGF-2, and EGF expression increase. Consistent with previous studies (25, 26), we confirmed that ECs and neural cells expressed L-PGDS in the P8 retina. Several inflammatory stimuli, such as cytokines and chemical substances, are known to upregulate L-PGDS expression in ECs (14, 27). Notably, the hypoxia niche accelerates L-PGDS-derived PGD_2_ production in rat atria by activating hypoxia-inducible factor 1α (28). According to a study showing that the neonatal retina is hypoxic until P8 and changes to normoxic conditions by P14 (29). We can thus conclude that L-PGDS is a regulator for producing appropriate pro-angiogenic cytokines to maintain normal angiogenesis under hypoxic conditions. Unexpectedly, vessel formation was normalized on P14, even under L-PGDS deficiency. Several compensatory systems normalize excessive angiogenesis. Angiopoietin 1 (Ang 1) and its receptor (TIE2) signaling stabilize ECs by suppressing the transcription of angiogenic factors (30). Unc-5 Netrin Receptor B (UNC5B) and Roundabout Guidance Receptor 4 (Robo4) signaling also stabilizes angiogenesis by inhibiting VEGF signaling in the murine retina (31). These systems might reverse L-PGDS-mediated inhibition of abnormal angiogenesis under normoxic conditions.

Our in vitro assays showed that the pretreatment of PGD_2_ metabolite, 15d-PGJ_2_ mediated the effect of L-PGDS in inhibiting abnormal angiogenesis. Although 15d-PGJ_2_ is known to upregulate VEGF-A expression, Jozkowics *et al.* showed that 15d-PGJ_2_ reduces VEGF-A expression by inhibiting hypoxia-inducible factor 1α under only hypoxic condition (32). Considering that both DP1 and DP2 deficiency did not affect the angiogenesis in P8 mice, 15d-PGJ_2_ may be involved in L-PGDS-induced neonatal retinal angiogenesis. However, in retinal angiogenesis, VEGF-A is mainly produced and regulated by astrocytes rather than ECs (23). Further investigations are needed to reveal the detailed mechanisms of L-PGDS-regulated normal angiogenesis.

Also 15d-PGJ_2_, PGD_2_ is metabolized into various prostaglandins, including 15-keto-PGD_2_ (13,14-dihydro-15-keto PGD_2_), Δ^12^-PGD_2_, 15d-PGD_2_ (15-deoxy-Δ^12,14^-PGD_2_), 9α,11β-PGF_2α_, and Δ^12^-PGJ_2_. Studies have shown that 15-keto-PGD_2_, Δ^12^-PGD_2_, 15d-PGD_2_, 9α,11β-PGF_2α_, and Δ^12^-PGJ_2_ can work as ligands of the DP2 receptor (33, 34). However, we previously showed that the treatment of DP2 agonism by 15-keto-PGD_2_ did not affect the angiogenesis in H-PGDS-deficient mice cornea (35). Additionally, 15-keto-PGD_2_ did not change endothelial barrier of HUVECs indicated as transendothelial resistance (36). In this study, DP2 deficiency did not affect the physiological angiogenesis either. These findings suggest that DP2 ligands may not be important for the physiological angiogenesis.

Unlike L-PGDS, H-PGDS deficiency did not affect physiological angiogenesis but did affect pathological angiogenesis. H-PGDS is expressed in hematopoietic cells, such as mast cells and macrophages. We found that H-PGDS was expressed in the infiltrated macrophages but not in the constituent cells, such as ECs and neural cells. The inducible expression of H-PGDS limits its functional role in physiological retinal angiogenesis.

PC depletion-induced retinopathy model produces clinical features of diabetic retinopathy, such as vascular tortuosity and dilation, edema, and hemorrhage (6). We found that H-PGDS-deficient retina, but not L-PGDS, increased vessel elongation compared with APB5-induced WT retina. H-PGDS deficiency also promoted retinal inflammation, especially hemorrhage and edema formation, as is shown from the results of retinal grading score. Our previous study showed similar results that, in the tumor implantation model, H-PGDS deficiency promotes vascular hyperpermeability and subsequent angiogenesis in implanted carcinomas (35). In the LPS-induced acute lung injury model and complete Freund’s adjuvant-induced joint inflammation model, H-PGDS deficiency increased the levels of inflammatory cytokines, including TNFα, IL-1β, and SDF1α (37, 38). Considering the H-PGDS expression in CD68-positive macrophages, the macrophages-derived H-PGDS might reduce PC depletion-induced retinal inflammation by decreasing vascular permeability or inflammatory cytokines. This signaling enhancement can be a new therapeutic tool for diabetic retinopathy.

We also found that DP1 deficiency, but not DP2, inhibited APB5-induced decrease of vessel elongation. We previously showed that DP1 receptor stimulation stabilizes the endothelial barrier through cAMP-PKA signaling (15). Treatment with a DP1 agonist decreased the mRNA levels of pro-angiogenic cytokines in macrophages (39). Thus, in the APB5-induced retinopathy model, H-PGDS/PGD_2_/DP1 signaling might downregulate retinal inflammation via endothelial barrier enhancement and/or macrophage cytokine expression decrease. In addition, we cannot exclude the contribution of PPARγ signaling activated by 15d-PGJ_2_. Jiang *et al.* reported that treatment with 15d-PGJ_2_ inhibited TNFα and IL-1β expression in monocytes (40). Further investigation is required to reveal whether DP receptor signaling and/or PPARγ signaling plays a crucial role in decreasing pro-angiogenic cytokine expression during inflammation.

Here, we focused on the differences in the expression, receptive signaling, and pathophysiological activities to reveal the significance of L-PGDS and H-PGDS in the retina. Each enzyme regulates normal retinal angiogenesis depending on its expression site, activation period, and mediated receptor signaling. However, angiogenesis also plays a crucial role in several situations, such as cancer growth, rheumatoid arthritis, and tissue repair. Differences in the type of activating cells and their period complicate clarifying the mechanism of angiogenesis in each situation. Our study thus provides new insights by focusing on each enzyme in multiple models.

## Data Availability

The data that support the findings of this study are available in the methods and/or supplementary material of this article.

## Supplemental data

This article contains supplemental data.

## Conflict of interest

The authors declare that they have no conflicts of interest with the contents of this article.
