# Supplementary material for: Roles of lipocalin-type and hematopoietic prostaglandin D synthases in mouse retinal angiogenesis
Source: J Lipid Res. 2023 Sep 4;64(10):100439. doi: 10.1016/j.jlr.2023.100439 (PMC10571029; doi:10.1016/j.jlr.2023.100439)
Supplement: Supplemental data [file mmc1.docx]

Roles of Lipocalin-type and Hematopoietic Prostaglandin D Synthases in Mouse Retinal Angiogenesis

Daiki Horikami^1^†, Erika Sekihachi^1^†, Keisuke Omori^1^, Yui Kobayashi^1^, Koji Kobayashi^1^, Nanae Nagata^1^, Kaori Kurata^2^, Tatsuro Nakamura^1^, Akiyoshi Uemura^2^, and Takahisa Murata^1^*

**Supplementary figures**


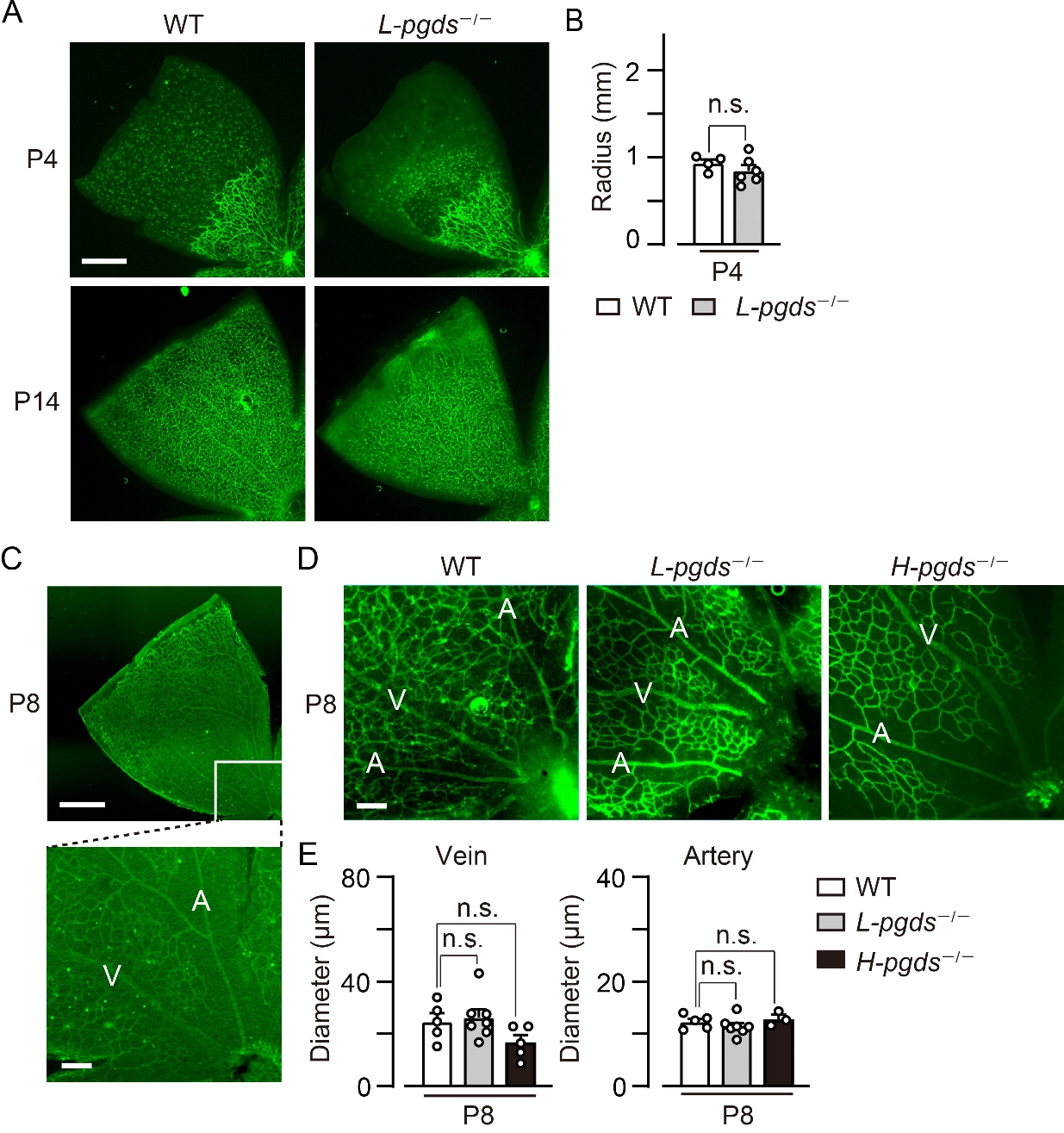


**Fig. S1.** **The effect of L-PGDS and H-PGDS deficiency on neonatal retinal angiogenesis.** P4, P8, and P14 retina of WT, *L-pgds*^-/-^, and *H-pgds*^-/-^ mice were excised, and ECs were stained with isolectin B4 (green). (A) Representative picture of the retina (n = 4-6). Scale bar, 500 µm. (B) The summary of vessel elongation (n = 4-6). (C) Picture of the P8 WT retina (upper panel; scale bar, 500 μm) and the base of the retinal vessel (high power field, lower panel; scale bar, 100 μm) A, artery. V, vein. (D) Representative pictures of the base of the retinal vessel (n = 3-7). A, artery. V, vein. Scale bar, 100 μm. (E) The summary of the diameter of vein and artery (n = 3-7). Data were represented as mean ± SEM. *P < 0.05. n.s., not significant.


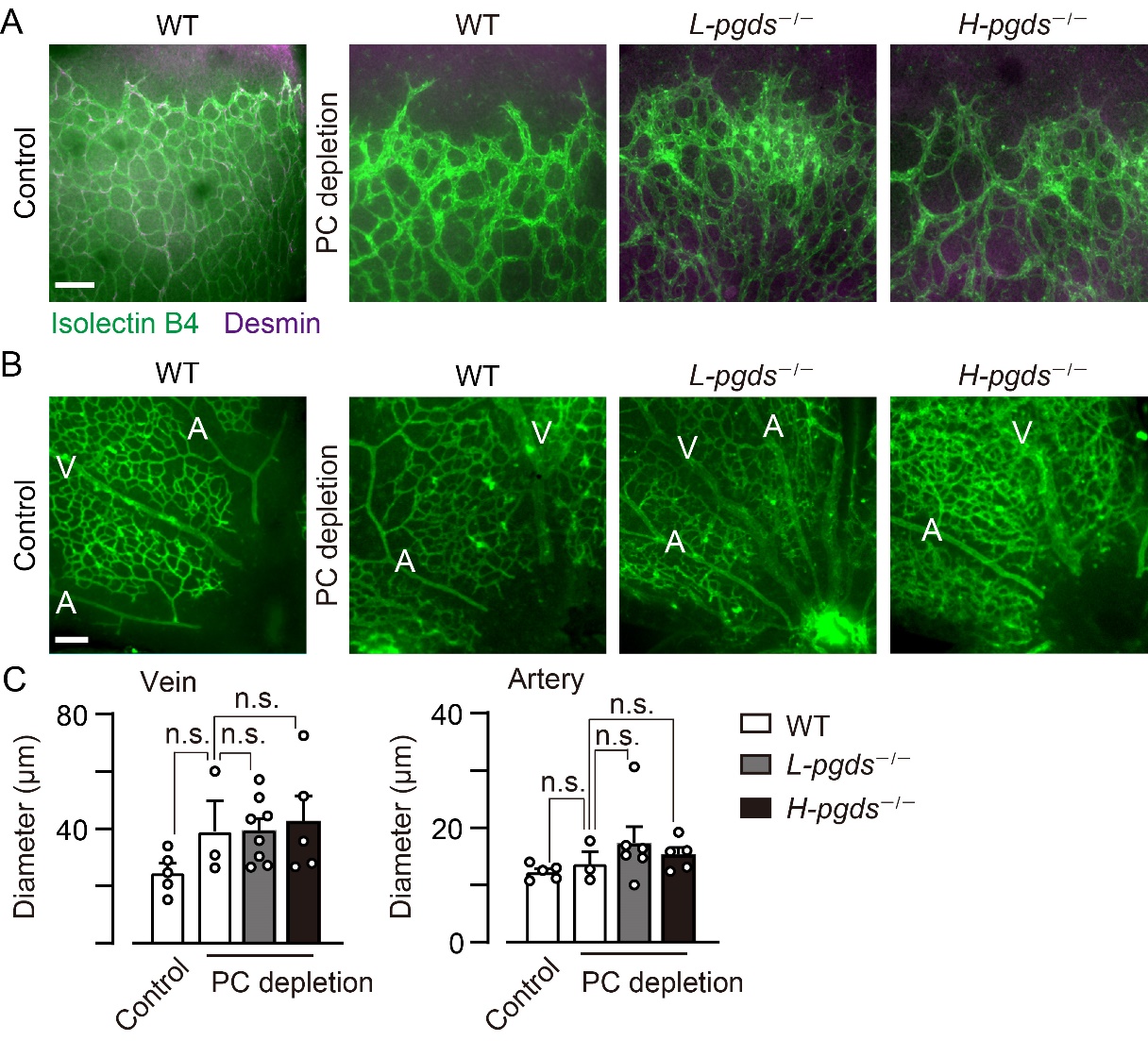


**Fig. S2.** **The effect of L-PGDS and H-PGDS deficiency on PC depletion-induced pathological angiogenesis.** After the PC depletion on P1 WT, *L-pgds*^-/-^ mice, and *H-pgds*^-/-^ mice, the P8 retina was excised and stained with isolectin B4 (green). (A) Representative pictures of the front of the retinal vessel stained with isolectin B4 and desmin antibody (magenta, n = 4-8). Scale bar, 100 μm. (B) Representative pictures of the base of the retinal vessel (n = 3-8). A, artery. V, vein. Scale bar, 100 μm. (C) The summary of the diameter of vein and artery (n = 3-8). The data of the WT control group is identical to Fig. S1E WT P8 group. Data were represented as mean ± SEM. n.s., not significant.


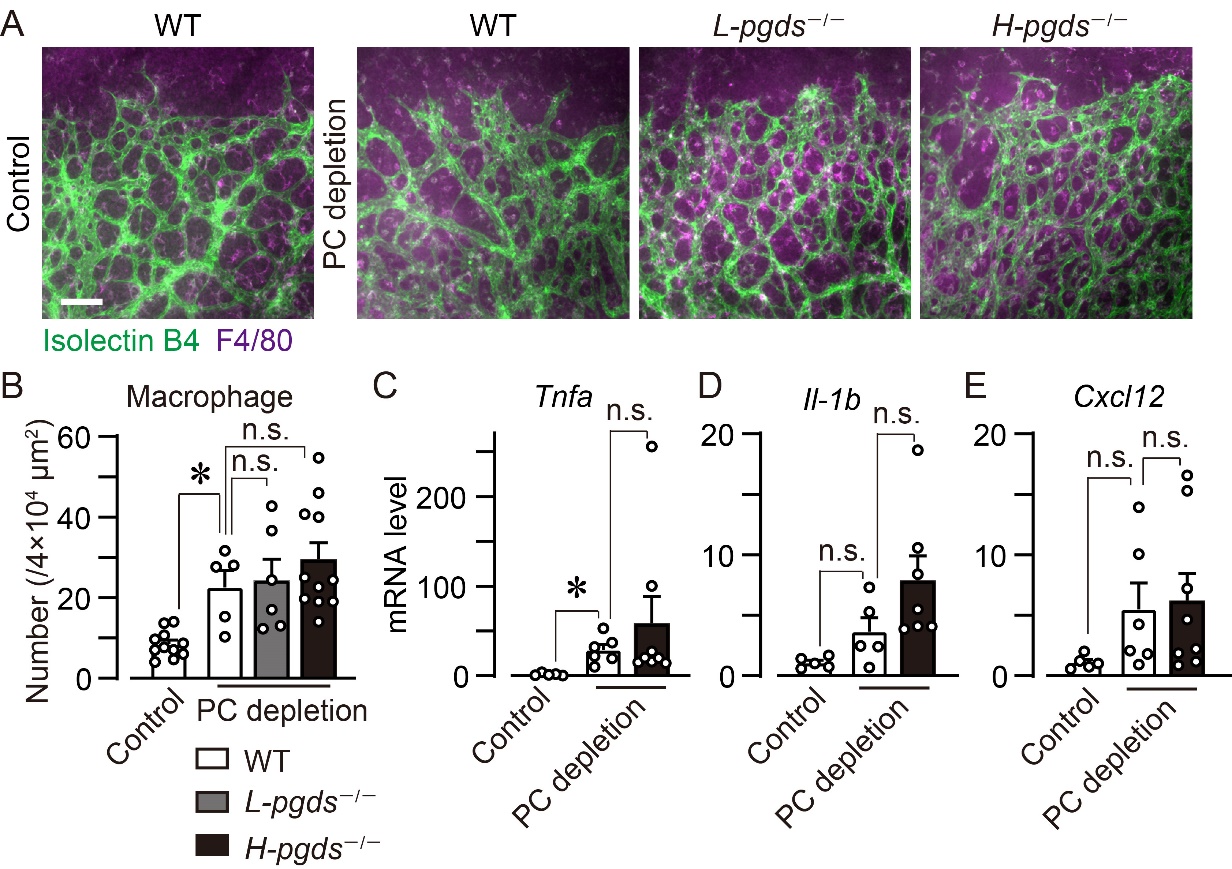


**Fig. S3.** **The macrophage infiltration and the mRNA expression of inflammatory cytokines effect in PC depletion-induced pathological angiogenesis.** After the PC depletion on P1 WT, *L-pgds*^-/-^ mice, and *H-pgds*^-/-^ mice, the P8 retina was excised and used for immunostaining and real-time PCR analysis. (A) Representative pictures of the front of the retinal vessel stained with isolectin B4 (green) and F4/80 (magenta, n = 3-5). Scale bar, 100 μm. (B) The summary of the number of F4/80-positive macrophages in the retinal area (n = 5-11). (C-E) The summary of mRNA level of (C) TNFα (*Tnfa*; WT control vs. WT PC depletion, p = 0.02; WT vs. H-PGDS, p = 0.96; n = 5-7), (D) IL-1β (*Il1b*; WT control vs. WT PC depletion, p = 0.17; WT vs. H-PGDS, p = 0.20; n = 5-7), and (E) CXCL12 (*Cxcl12*; WT control vs. WT PC depletion, p = 0.10; WT vs. H-PGDS, p = 0.96; n = 5-7). Data were represented as mean ± SEM. *P < 0.05. n.s., not significant.

**Supplementary tables:**

| Gene | Forward/  Reverse | Sequence 5' to 3' |
| --- | --- | --- |
|  |  |  |
| *Rn18s* | Forward | GACTCAACACGGGAAACCTCAC |
|  | Reverse | CACCCACGGAATCGAGAAAG |
| *L-pgds* | Forward | ACGAGGAGAGAGGGTCATCT |
|  | Reverse | GACACACTCTCCTGCTCAGT |
| *H-pgds* | Forward | TGGGAAGACAGCGTTGGAG |
|  | Reverse | AGGCGAGGTGCTTGATGTG |
| *Dp1* | Forward | CGCAAAACCAGAGCCTAAAG |
|  | Reverse | GCCATGAGGCTGGAGTAGAG |
| *Dp2* | Forward | CAAACCACAGCAACTCTAGC |
|  | Reverse | AGAAGACCGAGGAATGTAGC |
| *Vegfa* | Forward | GCTGTAACGATGAAGCCCTG |
|  | Reverse | CCTATGTGCTGGCTTTGGTG |
| *Fgf2* | Forward | CTTCTTCCTGCGCATCCATC |
|  | Reverse | TGGCACACACTCCCTTGATA |
| *Egf* | Forward | AGGGTGAACAAGAGGACTGG |
|  | Reverse | TTGGCAGATGTGTTCACAGC |
| *Vegfr2* | Forward | CCTCAATCTCACCTCTACCTTCC |
|  | Reverse | TCATAGTTGGCCTCCACCAC |
| *Il1b* | Forward | CAGGCAGGCAGTATCACTCA |
|  | Reverse | TGTCCTCATCCTGGAAGGTC |
| *Tnfa* | Forward | AGCCTGTAGCCCACGTCGTAG |
|  | Reverse | GTAGACAAGTGACAACCCATCG |
| *Sdf1a* | Forward | GCTCTGCATCAGTGACGGTA |
|  | Reverse | AGATGCTTGACGTTGGCTCT |

**Table S1. The primer list for murine retina**

| Gene | Forward/  Reverse | Sequence 5' to 3' |
| --- | --- | --- |
|  |  |  |
| *Rn18s* | Forward | CGTTCTTAGTTGGTGGAGCG |
|  | Reverse | AACGCCACTTGTCCCTCTAA |
| *Vegfa* | Forward | CGCAAGAAATCCCGGTATAA |
|  | Reverse | AAATGCTTTCTCCGCTCTGA |
| *Vegfr2* | Forward | ACGCTGACATGTACGGTCTAT |
|  | Reverse | GCCAAGCTTGTACCATGTGAG |

**Table S2. The primer list for HUVECs**
